# Antimicrobial Resistance of Clinical and Commensal *Escherichia coli* Canine Isolates: Profile Characterization and Comparison of Antimicrobial Susceptibility Results According to Different Guidelines

**DOI:** 10.3390/vetsci9060284

**Published:** 2022-06-09

**Authors:** Vera Fernandes, Eva Cunha, Telmo Nunes, Elisabete Silva, Luís Tavares, Luísa Mateus, Manuela Oliveira

**Affiliations:** 1 CIISA—Centro de Investigação Interdisciplinar em Sanidade Animal, Faculdade de Medicina Veterinária, Universidade de Lisboa, Av. da Universidade Técnica de Lisboa, 1300-477 Lisbon, Portugal; vm.fernandes@campus.fct.unl.pt (V.F.); tnunes@fmv.ulisboa.pt (T.N.); elisabetesilva@fmv.ulisboa.pt (E.S.); ltavares@fmv.ulisboa.pt (L.T.); lmateus@fmv.ulisboa.pt (L.M.); moliveira@fmv.ulisboa.pt (M.O.); 2 Laboratório Associado para Ciência Animal e Veterinária (AL4AnimalS), 1300-477 Lisbon, Portugal

**Keywords:** antimicrobial resistance, evaluation criteria, *Escherichia coli*, pyometra

## Abstract

Background: Pyometra is a diestrual chronic disease frequently associated with *Escherichia coli*. Initial pyometra treatment involves empiric antimicrobial therapy whose suitability should be confirmed by antimicrobial susceptibility testing. Antimicrobial resistance is a major health issue for veterinary medicine, rendering surveillance studies essential. Our goal was to determine the susceptibility profile of *E. coli* isolates obtained from healthy and pyometra-presenting dogs and to compare the application of different antimicrobial susceptibility guidelines. Methods: The antimicrobial susceptibility profile (ASP) of 74 *E. coli* isolates was determined by disk diffusion, using six antimicrobials commonly used in veterinary medicine. Profiles were assessed by CLSI VET01S, CLSI M100 and EUCAST guidelines. β-lactamases-encoding genes *bla*_TEM_, *bla*_SHV_ and *bla*_OXA_ were detected by multiplex PCR. Biofilm production ability was evaluated by pellicle formation assays in Luria–Bertani medium. Results: Variations in the resistance frequency were observed for amoxicillin/clavulanic acid, cephalexin and cefotaxime (29.7–54.1%, 10.8–16.2% and 1.4–4.1%, respectively). Results varied slightly between clinical and commensal isolates, as well as their biofilm-forming ability. Genes *bla*_TEM_, *bla*_SHV_ and *bla*_OXA_ were detected in 25.5%, 11.8% and 9.8% of isolates, respectively. Conclusions: Results show the importance of ASP determination in veterinary isolates and the need for using standardized and validated testing methods and harmonized interpretive criteria.

## 1. Introduction

Antimicrobial resistance (AMR) is the main undesirable side effect of antimicrobial abuse and misuse in both human and veterinary medicine, making it a major public health threat, as infections by antimicrobial resistant pathogens are associated with poor clinical outcomes [1,2]. With prevalent and novel mechanisms of resistance constantly evolving, the levels of complexity of resistance mechanisms exhibited by the pathogens are increasing, often resulting in therapeutic failure of first-line treatments [3,4]. In addition to the development of new antimicrobials, other measures are needed to control AMR.

Antimicrobial resistance surveillance is essential for acknowledgement of the complexity of AMR and for accessing the necessary information for implementing proper actions at the local, national and global levels [5]. It allows the identification of trends in pathogen and antimicrobial resistance incidence, including identification of emerging pathogens, identifying priority areas for interventions and monitoring the impact of those interventions [6,7]. Routine surveillance is critical for creating and refining approaches to control antimicrobial resistance and guide treatment choices [6,7]. However, there are still significant gaps in the surveillance of many bacterial pathogens that cause common infections, especially regarding companion animals. In fact, when compared to production animals, the antimicrobials administered to companion animals are more similar to those applied in human medicine; therefore, resistance development in this group may potentially have a more significant impact on public health [8,9]. As such, it is of extreme importance to minimize and rationalize the use of antimicrobials in companion animals without affecting their clinical efficacy, in a prudent and rational manner, but avoiding impairment of the clinical outcome. To do so, antimicrobial susceptibility testing (AST) is essential, as it predicts the resistance profile of the pathogen involved in the infectious disease and helps in the selection of the most appropriate drug for a specific treatment [10]. Breakpoints for phenotypic AST are developed and allow the categorization of the activity of a drug against the isolated organism [11]. 

The Clinical Laboratory Standards Institute (CLSI) and the European Committee on Antimicrobial Susceptibility Testing (EUCAST) guidelines are the most popular breakpoint guidelines worldwide, EUCAST being largely used in Europe [12]. However, it still does not have clinical breakpoints established for testing veterinary isolates. Standardization of breakpoint guidelines and methods can ensure consistent clinical reporting of AST results and comparable data in antimicrobial resistance surveillance [12]. Given the current concerns about the emergence and trends in antimicrobial resistance dissemination in bacteria of animal origin, there is an urgent need for reliable interpretative criteria for optimum and controlled antimicrobial prescriptions, this requirement being dependent on the surveillance of veterinary pathogens, for which specific guidelines are still insufficient [13].

Among the most common pathogens in veterinary medicine is *Escherichia coli*, a commensal microorganism present in the gastrointestinal tract of animals that is capable of causing several diseases, both intestinal and extraintestinal, using a variety of pathogenic mechanisms [14]. The emergence of antimicrobial resistance is threatening the therapeutic treatment of *E. coli*-related infections, with the prevalence of multidrug-resistant *E. coli* strains increasing worldwide [14].

Pyometra is one of the main uterine diseases diagnosed in bitches, affecting approximately 25% of ovary-intact female canines before 10 years of age [15,16]. It is a diestrual chronic disease with multifactorial pathogenesis, being the presence of high progesterone levels a keystone in disease development [15]. Pyometra is associated with severe bacterial infection, being *E. coli* the most prevalent isolated microorganism [14,15]. Consequently, bitches may develop acute clinical signs that can be potentially life threatening if no action is taken [14,15].

The treatment of pyometra should be established immediately after diagnosis, involving empiric antimicrobial therapeutics independently if medical or surgical treatments aiming at controlling progesterone levels and emptying the uterus are implemented [15,16]. In many cases, other infections caused by the same microorganism, such as urinary tract infections or peritonitis, may be present, reinforcing the importance of antimicrobial therapy in these animals [16]. However, it is important to note that the use of antimicrobials in association with medical treatment may not be fully effective, being ovariohysterectomy the most recommended treatment option [15]. Some of the possible reasons are the inability of antimicrobials to diffuse into the pool of intrauterine fluid and possible biofilm formation [15]. Biofilms are microbial communities enclosed in an adherent extracellular polymeric matrix constituted by polysaccharides, proteins and nucleic acids [17]. Microorganisms undergo significant changes during the transition from planktonic to biofilm growth, involving metabolic, physiological and phenotypic changes, coordinated by intracellular signaling pathways in response to environmental changes, thereby permitting the adaption to unfavorable conditions [18,19].

Biofilm formation is associated with the chronic nature of some infections due to their inherent resistance to antimicrobial therapy [20]. Bacteria in biofilms are more resistant to antimicrobials and evade more easily the hosts’ immune system defense mechanisms [20,21]. The reason is not fully understood, but it appears that this higher tolerance to antimicrobials is due to the co-working of multiple factors [21]. In addition to biofilm production, there are several other mechanisms associated with antimicrobial resistance in Gram-negative bacteria, being the production of β-lactamases one of the most important ones, as it is also the principal mechanism of resistance to β-lactam antimicrobials, a group, which includes some of the most frequently prescribed antimicrobials worldwide [2,22]. β-lactamases are remarkably diversified due continuous mutations, with more than 400 β-lactamases having been documented [22,23]. TEM, SHV and OXA type are among the most common classes of β-lactamases in companion animals. As many clinical isolates now harbor more than one β-lactamase gene and due to the high diversity of β-lactamases, multiplex PCR assays are becoming widely used for their detection in epidemiological surveys, as they confer a more rapid approach to identifying several classes, allowing the detection of more than one target in a single PCR reaction [23].

The main purpose of this study was to determine the susceptibility profile of *Escherichia coli* isolates obtained from healthy and pyometra-presenting dogs and to compare the application of different antimicrobial susceptibility guidelines. The detection of biofilm formation capability and of β-lactamases-encoding genes *bla*_TEM_, *bla*_SHV_ and *bla*_OXA_ were also performed.

## 2. Materials and Methods

### 2.1. Bacterial Strains

In this study, a collection of 74 *Escherichia coli* isolates obtained from healthy (n = 41) and pyometra-presenting (n = 33) dogs were used as bacterial models. All isolates were obtained using sterile swabs during diagnostic activities in the Teaching Veterinary Hospital at the Faculty of Veterinary Medicine, University of Lisbon [24,25]. Pyometra isolates were recovered from uterine swabs of bitches with pyometra, after ovariohysterectomy [24]. Fecal isolates were obtained from healthy bitches with no clinical history of pyometra or urinary tract infections (UTI) [24]. The isolates’ phylogenetic group was determined in previous studies by multiplex PCR [24,25]. Isolates were cryopreserved in buffered peptone water plus 20% of glycerol and kept at −80 °C during this study.

### 2.2. Antimicrobial Susceptibility Testing

In order to analyze the suitability of the criteria established by three of the most used antimicrobial susceptibility guidelines, namely CLSI VET01S, CLSI M100 and EUCAST, when applied to isolates of animal origin, antimicrobial susceptibility testing (AST) using the disc diffusion method was performed [13,26,27]. The disc diffusion tests were performed for each strain using six antimicrobials frequently used in veterinary medicine (OXOID, Hampshire, UK): amoxicillin/clavulanic acid (AMC, 30 μg), ampicillin (AMP, 10 μg), enrofloxacin (ENR, 5 μg), cephalexin (CL, 30 μg), cefotaxime (CTX, 30 μg) and sulfamethoxazole/trimethoprim (SXT, 25 μg). 

Assays were performed according to CLSI VET01-A4 [28]. Afterward, susceptibility profiles of the isolates were assessed using CLSI VET01S, CLSI M100 and EUCAST guidelines, which allowed determining whether the organisms were susceptible, intermediate or resistant to each antimicrobial agent tested, according to the different susceptibility interpretative criteria [13,26,27]. An additional cefoxitin (30 μg) (OXOID, Hampshire, UK) disc diffusion test was performed regarding 18 of the 78 strains, in view of their resistance profile to ampicillin and amoxicillin/clavulanic acid, the synergy between the 2 antimicrobials and a susceptible profile to cefotaxime. The percentage of multidrug-resistant *Escherichia coli* strains was evaluated by considering as a multidrug resistance isolate a strain resistant to antimicrobials from three or more classes [29].

### 2.3. Biofilm Formation in the Air–Liquid Interface

In order to study the biofilm formation capacity of the *Escherichia coli* isolates, pellicle formation assays in Luria–Bertani (LB) medium were performed [30]. LB medium was prepared using Yeast Extract (OXOID, Hampshire, UK) and Bacto Tryptone (BD, New Jersey, EUA). Biofilm formation in the air–liquid interface was assessed by inoculation of 0.5 mL of an overnight culture in 4.5mL of LB medium without NaCl (1:10) in glass tubes, incubated at 28 °C for 8 days. Each isolate was visually examined every 24 h for biofilm formation. Biofilm formation phenotypes were associated with the formation of a pellicle containing a tight bacterial network on the air–liquid interface in LB medium. Three assays were performed for each isolate on separate days, and 10% of replicates were also tested. Biofilm formation in the air–liquid interface was considered positive when pellicle formation was observed in at least 2 independent assays.

### 2.4. Multiplex Polymerase Chain Reaction Assay of β-lactamases-Encoding Genes bla_TEM_, bla_SHV_ and bla_OXA_

A multiplex polymerase chain reaction (PCR) assay targeting genes encoding TEM, SHV and OXA β-lactamases was performed for the isolates expressing a phenotypic resistance profile to ampicillin and/or amoxicillin/clavulanic acid. Thus, 51 *E. coli* isolates (21 pyometra isolates and 30 fecal isolates) were included in this analysis. The multiplex PCR protocol used in this assay was adapted from a previously published protocol [23].

#### 2.4.1. DNA Extraction

Total DNA was extracted using a boiling procedure. To do so, 2 to 3 colonies were picked from pure cultures in Mueller–Hinton agar (OXOID, England), incubated overnight at 37 °C and suspended in 50 μL of 0.5× TE buffer complemented with 0.1% of Tween 20 (Merck, Lisbon, Portugal), followed by homogenization. TE buffer 0.5× was prepared using a 10× stock solution of TBE buffer (NZYTech, Lisbon, Portugal) with a 1:20 dilution in distilled water. Suspensions were incubated at 100 °C for 10 min, followed by a 5 min incubation on ice. Suspensions were then centrifuged at 11,250 rpm for 10 min. The DNA supernatant obtained by centrifugation was used immediately or stored at −20 °C until further use.

#### 2.4.2. PCR Amplification

The multiplex PCR assay was performed using primers previously described, synthetized by STABVIDA (Table 1) [23]. 

Each multiplex PCR mixture, with a final volume of 20 µL, contained 10 µL of Supreme NZYTaq 2x Green Master Mix (0.2 U/μL) (NZYTech, Lisbon, Portugal), 0.3 µL of each specific primer, 7.2 µL of sterile water and 1 µL of DNA. Positive controls and a negative control with no DNA were used in all PCR reactions. The positive controls for TEM, SHV and OXA-type genes were kindly provided by Dr Lurdes Clemente of Instituto Nacional de Investigação Agrária e Veterinária (INIAV, Oeiras, Portugal). Amplification was carried out in a thermal cycler (MyCycler Thermal Cycler, Bio-Rad, Lisbon, Portugal) using the following conditions: initial denaturation step at 94 °C for 10 min; 30 cycles consisting of denaturation at 94 °C for 40 s, annealing at 60 °C for 40 s and elongation at 72 °C for 1 min; and a final elongation step at 72 °C for 7 min. The amplicons were visualized by conventional electrophoresis in a 2% agarose gel (NZYTech, Lisbon, Portugal) with 0.5% Tris/Boric Acid EDTA (NZYTech, Lisbon, Portugal) buffer stained with GelRed (Biotium, Fremont, CA, USA) at 90 V for 1 h 10 min. NZYDNA ladder VI (NZYTech, Lisbon, Portugal) was included as a molecular weight marker. Results were visualized by transillumination under UV using the Molecular Imager^®^ Gel Doc™ XR System (Bio-Rad, Lisbon, Portugal) and Image LabTM Software (Bio-Rad, Lisbon, Portugal).

### 2.5. Statistical Analysis

SPSS version 20.0 for Windows (SPSS Inc., Chicago, IL, USA) was used for statistical analyses. Cohen’s kappa was used to measure the agreement between the susceptibility profile results obtained by the three different guidelines. The interpretation of κ in the interval (0.1) was analyzed, as suggested by Landis and Koch [31]. A possible relation between the resistance profile and the phylogenetic group of the strains and a possible relation between the antimicrobial susceptibility profile for each antimicrobial tested and the capability of biofilm formation were investigated using the Chi-square test, and a *p*-value ≤ 0.05 was considered statistically significant. Pearson correlation was used to measure a possible correlation between the resistance profile of the strains analyzed, measured as the number of antimicrobials tested defined as resistant, and the biofilm formation capacity, measured by the number of days until a certain isolate showed pellicle formation.

## 3. Results

### 3.1. Antimicrobial Susceptibility Testing

The susceptibility results for all the isolates under study are summarized in Table 2, organized considering the strains’ origin and AST guideline interpretation.

It is possible to note that there were differences in the susceptibility profile results (highlighted) when applying different antimicrobial susceptibility testing evaluation criteria. However, the susceptibility profiles determined based on CLSI M100 and CLSI VET01S standards are similar when AST evaluation criteria are present in both guidelines, which can be explained by the fact that the CLSI veterinary guideline includes human-derived breakpoints.

The frequency of resistance, as determined between different guidelines, was similar for ampicillin (59.5%), enrofloxacin (5.4%) and sulfamethoxazole/trimethoprim (10.8%); variations were observed for amoxicillin/clavulanic acid, cephalexin and cefotaxime (29.7–54.1%, 10.8–16.2% and 1.4–4.1%, respectively). There were slight variations in susceptibility profiles between the *Escherichia coli* clinical strains isolated from pyometra-presenting animals and commensal strains obtained from fecal samples of healthy animals. From the 74 isolates under study, the susceptibility of 18 isolates against cefoxitin was also tested. Strains presenting a reduced susceptibility to cefoxitin can be presumptively considered as producers of AmpC enzymes, and as such, screening of plasmid-mediated AmpC β-lactamases is recommended [32]. However, all 18 *Escherichia coli* strains were susceptible to cefoxitin, and therefore, no further PMAβ testing was conducted.

#### 3.1.1. Determination of Multidrug-Resistant Strains

None of the pyometra strains under study was considered MDR. However, the frequency of MDR strains among the commensal samples varied according to the antimicrobial susceptibility testing guideline considered, amounting to 7.32%, 9.76% and 2.44% when considering CLSI M100, CLSI VET01S and EUCAST version 6.0 standards, respectively. 

#### 3.1.2. Measurement of the Agreement between the Antimicrobial Susceptibility Testing Guidelines

**Figure 1:**
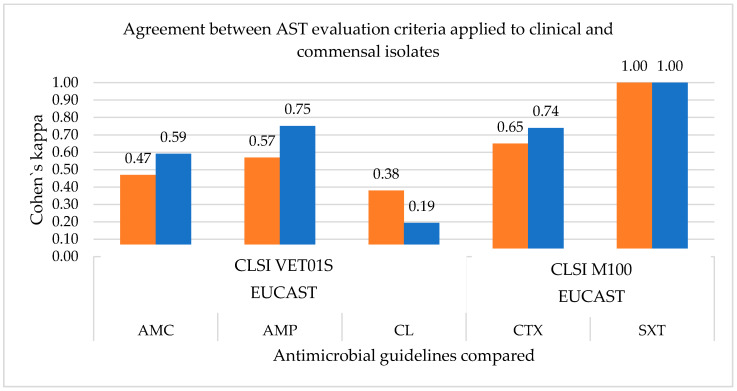
Measurement of the agreement between AST guidelines for the different antimicrobials tested. Cohen’s kappa takes values of (0.1). Clinical strains are represented in orange and commensals in blue.

Cohen’s kappa was used to measure the agreement between antimicrobial susceptibility profiles assessed between the AST guidelines. Since enrofloxacin is only used in veterinary medicine, there are no AST standards in human-related guidelines. Therefore, the susceptibility profile for this agent was only assessed by CLSI VET01S standards, and the measurement of agreement was not necessary. Additionally, as susceptibility profiles are equal when using CLSI M100 or CLSI VET01S for ampicillin, amoxicillin/clavulanic acid, cephalexin and sulfamethoxazole/trimethoprim, as human-derived breakpoints are used for these antimicrobial agents in CLSI VET01S standards, only the comparison between CLSI VET01S and EUCAST is shown in Figure 1. 

It is possible to note that there is perfect agreement in both pyometra and commensal strains for the sulfamethoxazole/trimethoprim susceptibility results when using CLSI VET01S and EUCAST version 6.0 evaluation criteria standards. However, this is not true for the remaining antimicrobials, with Cohen’s kappa value < 1. These differences in agreement between AST results when using different guidelines leads to differences in susceptibility profiles to the antimicrobials tested, which can lead to differences in therapeutic options.

### 3.2. Antimicrobial Resistance Profile and Phylogenetic Group

The clinical strains tested belong mainly to phylogenetic group B2 (97.0%), with only one strain (3.0%) belonging to group B1. As for the commensal strains, the phylogenetic group was more diverse, with strains belonging to group B2, B1, A and D, with frequencies of 43.9%, 19.5%, 12.2% and 24.4%, respectively. Most of the strains under study belong to phylogenetic group B2, which corresponds to 67.6% of the totality of samples. Strains belonging to B2 are generally more virulent and have a higher number of virulence determinants among the phylogenetic groups [33]. A possible relation between the resistance profile and the phylogenetic group of the strains was investigated using Pearson Chi-square test. No association between the phylogenetic groups and the number of resistances to the antimicrobials tested was observed (χ² = 18.821; *p*-value = 0.222).

### 3.3. Biofilm Formation in the Air–Liquid Interface

**Figure 2:**
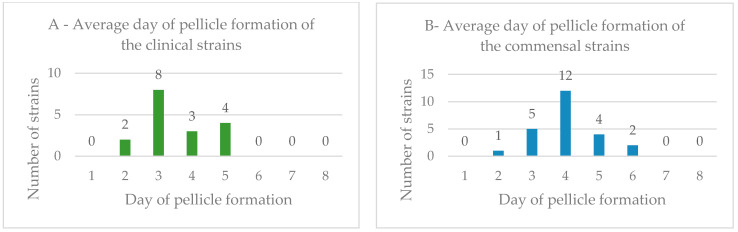
(**A**) Average day of pellicle formation in the air–liquid interface for the *Escherichia coli* clinical strains; (**B**) Average day of pellicle formation in the air–liquid interface for the *Escherichia coli* commensal strains from canines.

Biofilm formation capacity of the *E. coli* strains from pyometra-presenting and healthy bitches was analyzed using pellicle formation assays in Luria–Bertani medium. The frequency of biofilm formation among clinical and commensal isolates was 51.52% and 57.14%, respectively. The average day of pellicle formation among the *E. coli* strains, including clinical and commensal samples, is shown in Figure 2. 

For the positive clinical strains, the frequency of pellicle formation was 11.76% (day 2), 47.06% (day 3), 17.65% (day 4) and 23.53% (day 5). For the positive commensal strains, the frequency of pellicle formation was 4.17% (day 2), 20.83% (day 3), 50.00% (day 4), 16.67% (day 5) and 8.33% (day 6). The most prevalent days for biofilm formation were different between clinical and commensal strains, those being the third and fourth days, respectively.

### 3.4. Biofilm Formation and Antimicrobial Resistance Profile

Biofilm formation capability is a mechanism associated with an increase in tolerance to antimicrobials [34]. A possible correlation between the number of resistances to antimicrobials and the day of pellicle formation was investigated using the Pearson correlation test. No significant correlation was observed for the commensal strains (r = 0.088) nor for the clinical strains (r = −0.099). 

A possible relation between the strains’ antimicrobial susceptibility profile toward each antimicrobial tested and their capability of biofilm formation were investigated using the Pearson Chi-square test. No significant relation was found between the biofilm formation capacity of the strains under study and the susceptibility profile to each antimicrobial tested, namely amoxicillin/clavulanic acid (χ² = 0.397; *p*-value = 0.820); ampicillin (χ² = 0.242; *p*-value = 0.886); enrofloxacin (χ² = 0.588; *p*-value = 0.745); cephalexin (χ² = 4.796; *p*-value = 0.091); cefotaxime (χ² = 2.048; *p*-value = 0.359); and sulfamethoxazole/trimethoprim (χ² = 1.904; *p*-value = 0.386).

### 3.5. Multiplex Polymerase Chain Reaction Assay of βlactamases-Encoding Genes bla_TEM_, bla_SHV_ and bla_OXA_

**Figure 3:**
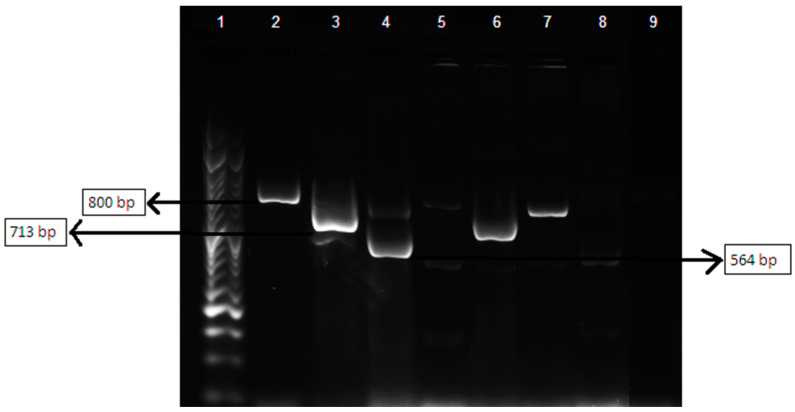
Amplification products of *bla*_TEM_, *bla*_SHV_ and *bla*_OXA_. Lane 1—NZYDNA Ladder VI; Lane 2—*bla*_TEM_ positive control; Lane 3—*bla*_SHV_; Lane 4—*bla*_OXA_-1 positive control; Lane 5 to 8—Amplification products of *E. coli* strains P1, P11, P13 and P14; Lane 9—Negative control.

The presence of the β-lactamases-encoding genes *bla*_TEM_, *bla*_SHV_ and *bla*_OXA_ was detected in 25.5%, 11.8% and 9.8% of the 51 strains tested, respectively. The frequency of *bla*_TEM_, *bla*_SHV_ and *bla*_OXA_ among clinical and commensal isolates differed, with 14.3%, 14.3% and 14.3%, respectively, in the clinical strains; and 33.3%, 10.0% and 6.7% in the commensal strains. In Figure 3, the positive controls for *bla*_TEM_, *bla*_SHV_ and *bla*_OXA,_ and the amplicons of strains P 1, P 11, P 13 and P 14 are shown.

With regard to the phylogenetic grouping of *E. coli* strains, strains belonging to the phylogenetic group B2 had the highest frequency of *bla*_TEM_, *bla*_SHV_ and *bla*_OXA_, with 53.9%, 66.7% and 100% of the totality of strains encoding these genes, respectively.

## 4. Discussion

The dissemination of antimicrobial-resistant bacteria is a serious threat to both public and animal health. Additionally, the proximity between household pets and humans and the use of important classes of antimicrobials for human health in companion animals increases the chances of interspecies transmission of resistant strains [34]. Since about 25% of the households in the European Union have at least one companion animal, this can be a serious issue if no measures are taken [35]. However, antimicrobial resistance surveillance programs focused on companion animals are not widespread, which is problematic, as the monitorization of antimicrobial trends among bacteria isolated from pets can be useful for guiding antimicrobial administration practices [36,37]. With the current concern about the emergence of resistant strains of pathogenic or commensal bacteria species from animals and their potential spread to humans, studies to access the susceptibility profile of veterinary isolates to antimicrobial agents are essential, allowing us to obtain valuable data aiming at controlling AMR dissemination to guide antimicrobials administration policies in small animals veterinary practice and to assess the risk factors associated with the emergence of multidrug-resistant bacteria and interspecies transmission of these strains [35,38].

*Escherichia coli* seems to be an ideal bacterial model for such studies, as it is one of the main pathogens in dogs, a versatile organism and a commensal inhabitant of the intestinal tract of both companion animals and humans [14,38]. *E. coli* has a high mutagenic capacity and is capable of rapid cell division, which makes it able to adapt to various environments and to the presence of antimicrobials compounds [39].

In this study, the susceptibility profile of *E. coli* strains from pyometra-presenting and healthy dogs was accessed, using antimicrobials which are among the most prescribed in the initial treatment of pyometra [15]. The interpretation of AST was performed using three of the most commonly used AST guidelines worldwide: CLSI M100 and EUCAST version 6.0, two guidelines developed for human medicine but sometimes adapted for veterinary medicine; and CLSI VET01S, an AST guideline for veterinary application.

Higher percentages of resistance to ampicillin and amoxicillin/clavulanic acid were observed. These profiles were expected, as these antimicrobials are highly used in the empirical treatment of pyometra and have been applied for longer in veterinary medicine than more recent antimicrobials or antimicrobial combinations, such as enrofloxacin or sulfamethoxazole/trimethoprim [40].

*E. coli* associated with canine pyometra usually originates from the normal intestinal microbiota of dogs, not being related to any specific clonal type, and is characterized by harboring several virulence genes usually found in uropathogenic strains (UPEC) [24,41]. The fact that *E. coli* strains associated with pyometra originate from commensal variants can, in part, explain the similar rates of resistance to the antimicrobials tested in this study. The resistance profiles of the commensal *E. coli* isolates from healthy dogs show that the intestinal tract of healthy animals can constitute a reservoir of resistant strains and their determinants [42]. However, regarding companion animals, there is still a lack of monitorization programs at regional, national and international levels, which are essential to guide policies and detect changes that require intervention strategies. As multidrug-resistant *E. coli* is capable of expressing resistance determinants, such as production of β-lactamases, and can be isolated from both healthy animals and clinical cases, the surveillance of both types of strains is of vital importance [36].

In this study, it was possible to observe that the susceptibility profile of the strains varied when using different AST interpretation criteria. This can constitute a serious problem not only in the selection of the antimicrobial therapeutic protocol but also in the surveillance of antimicrobial resistance, as differences in data can compromise their comparison. In order to measure the degree of agreement between AST results when using different standards, Cohen‘s kappa was calculated. As previously mentioned, the susceptibility profiles were similar when using CLSI M100 or CLSI VET01S evaluation criteria for ampicillin, amoxicillin/clavulanic acid, cephalexin and sulfamethoxazole/trimethoprim for *Enterobacteriaceae*, as human-derived breakpoints are used in CLSI VET01S standards. The use of such breakpoints constitutes a serious problem in the AST determination of veterinary isolates, as the differences in pharmacodynamics (PD) and pharmacokinetics (PK) between humans and other animals are not considered [43]. PK-PD studies are used in AST breakpoint development, as they describe the relationship between the effect of the antimicrobial on the infecting organism, typically measured in vitro (pharmacodynamics), and clinical and microbiological outcomes in the host, measured in vivo (pharmacokinetics) [44].

Regarding the measured agreement between CLSI VET01S and EUCAST version 6.0 evaluation criteria standards, although Cohen’s kappa revealed a perfect agreement regarding sulfamethoxazole/trimethoprim for the *E. coli* strains under study, this was not observed for the remaining antimicrobials tested. In these cases, their Cohen’s kappa value was <1, ranging from 0.47–0.59 for AMC, 0.57–0.75 for AMP, 0.19–0.38 for CL and 0.65–0.75 for CTX, among the clinical and commensal isolates. This fact may be explained by the differences in the breakpoint setting process between the two AST agencies that lead to different interpretative criteria values for the same antimicrobial agent. Breakpoints setting is based on clinical, pharmacokinetic, microbiological and pharmacodynamic studies; however, the relative importance of each parameter is under constant discussion, and so, AST agencies can adopt different approaches [45]. CLSI and EUCAST are the two main nongovernmental agencies in setting breakpoints standards for antimicrobial susceptibility testing [46]. However, these agencies differ in their approach to breakpoints setting, internal organization, data collection establishment and also in their report obligations to their governmental counterparts, explicitly the U.S. Food and Drug Administration (FDA) in the case of CLSI and the European Medicines Agency (EMA) in the case of EUCAST [44,46]. Other differences include the role of industry, funding and the availability of the standards developed, as EUCAST standards are freely available while CLSI are not [44,46]. As previously mentioned, setting or reviewing clinical breakpoints currently takes into consideration a combination between pharmacokinetic and pharmacodynamic data, clinical outcome data and minimum inhibitory concentrations (MIC) in vitro data [47]. With breakpoints for AST being expressed in either concentration or zone diameter, depending on the testing method applied, and disk diffusion being one of the most widely used AST methods in routine clinical microbiology laboratories, there is a need to have zone diameter breakpoints calibrated considering the MIC breakpoints [48]. This is particularly relevant in Europe, where a harmonizing process of AST methodologies was carried out by EUCAST on European MIC breakpoints [48]. Regarding AST in veterinary medicine, few species-specific interpretative breakpoints are available regarding MIC, and to an even greater extent, disk diffusion breakpoints. Particularly in this study, when analyzing the clinical breakpoints available in CLSI-VET01S for *Enterobacteriaceae*, the bacterial family to which *E. coli* belongs, it was possible to note that there were no species-specific disk diffusion breakpoints for the studied antimicrobials, except for enrofloxacin, for which disk diffusion breakpoints applicable to *Enterobacteriaceae* strains isolated from dogs are available. As such, the development of appropriate clinical breakpoints, both for MIC and disk diffusion for veterinary isolates is vital for predicting treatment success of infections in companion animals and guiding clinicians in the selection of the most adequate therapeutics [49]. 

To further characterize the *E. coli* strains under study in terms of resistance, biofilm production ability was accessed, as well as the presence of β-lactamases genes *bla*_TEM_, *bla*_SHV_ and *bla*_OXA_, as biofilm production is a potential virulence trait, and the production of β-lactamases is the main mechanism of resistance to β-lactams in *Enterobacteriaceae* [2,50]. The biofilm formation capacity of the *E. coli* strains from pyometra-presenting and healthy animals was analyzed using pellicle formation assays, with approximately half of the clinical strains and of the commensal isolates showing a biofilm formation phenotype; however, the majority of the clinical strains were able to form biofilms within a three-day period, while most of the commensal strains required a four-day period to form biofilms, meaning that the *E. coli* strains from pyometra-presenting dogs, on average, displayed a more rapid biofilm formation process than the commensal isolates. Lopes et al. (2021) also evaluated the biofilm ability of *E. coli* isolates obtained from canine pyometra, observing a high prevalence of positive biofilm strains, reinforcing the importance of this virulent element in infection [51]. It would be interesting to test biofilm-forming ability in these pyometra isolates, simulating the conditions in the uterus of canines suffering from this disease, as in vivo conditions may influence this virulent factor [51].

Another important resistance determinant in *E. coli* is β-lactamases production, being one of the main mechanisms of resistance to β-lactam antimicrobials among *Enterobacteriaceae* in both human and veterinary medicine but also frequently associated with resistance to several other groups of antimicrobials, including fluoroquinolones, aminoglycosides or sulfamethoxazole [30,52]. Since companion animals are a potential reservoir of β-lactamases-encoding genes, and their close contact with their owners facilitates resistance transmission, surveillance studies to detect the prevalence of β-lactamases-encoding genes are important and needed in veterinary medicine [53,54]. Multiplex PCR approaches are suitable for the rapid detection of the emergence of β-lactamases in bacteria isolated from animals, improving surveillance [23]. In a β-lactamase family type, the resistance phenotypes expressed can differ substantially among enzyme members due to changes in the enzyme structure and activity [55]. In fact, in this study, the strains identified as having β-lactamase-related genes of a certain β-lactamase family (e.g., TEM, SHV or OXA) showed different susceptibility profiles to the antimicrobials tested.

The identification of the β-lactamase(s) produced by the strains using a sequencing technique and the antimicrobial susceptibility testing of more β-lactams would provide extra knowledge of the specific resistance pattern. Furthermore, these enzymes are often plasmid mediated, which can facilitate their transmission among individuals and their dissemination in the environment. Thus, the presence of β-lactamases-related genes in the clinical and commensal *E. coli* strains under study, particularly plasmid-mediated β-lactamases, is of concern, as their transmission can occur frequently. 

## 5. Conclusions 

Antimicrobial-resistant bacteria are a serious threat to human and animal health. Several AST guidelines are available for the evaluation of the antimicrobial-resistant signatures in human strains; however, specific guidelines for AMR evaluation of animal-origin isolates and specific breakpoints are scarce. In this study, an antimicrobial susceptibility profile characterization of canine *E. coli* clinical and commensal isolates was performed. Results showed the importance of determining the antimicrobial resistance profile of veterinary isolates and the need for the implementation of national and international surveillance programs for companion animals. The low agreement between AST results when applying different guidelines to certain antimicrobials largely used in companion animals and the use of human-derived breakpoints can constitute a serious problem, possibly leading to poor clinical outcomes and resistance dissemination. In view of this, it is urgent to have standardized and validated testing methods and harmonized interpretive criteria in veterinary medicine, enabling the identification of multidrug-resistant bacteria, as they represent a clinical challenge for domestic animal medicine. Additionally, the presence of β-lactamases-related genes in the strains under study, and particularly plasmid-mediated β-lactamases, is of concern, as their transmission occurs frequently. Furthermore, biofilm-forming ability being related to bacterial virulence, its association with antimicrobial resistance determinants is of great concern, particularly in companion animals due to close contact with humans, possibly acting as a reservoir of bacteria-harboring resistance genes. 

## Figures and Tables

**Table 1:** Nucleotide sequences of the primers used for the amplification of *bla*_TEM_/*bla*_SHV_/*bla*_OXA_ genes and size of the respective amplicons.

β-lactamases Targeted	Sequence (5′–3′)	Product Size (bp)
TEM variants	F_CATTTCCGTGTCGCCCTTATTC	800
	R_CGTTCATCCATAGTTGCCTGAC	
SHV variants	F_AGCCGCTTGAGCAAATTAAAC	713
	R_ATCCCGCAGATAAATCACCAC	
OXA-1, OXA-4 and OXA-30	F_GGCACCAGATTCAACTTTCAAG	564
	R_GACCCCAAGTTTCCTGTAAGTG	

**Table 2:** Antimicrobial susceptibility testing results for ampicillin, amoxicillin/clavulanic acid, enrofloxacin, cephalexin, cefotaxime and sulfamethoxazole/trimethoprim. The frequency of resistant, intermediate and susceptible strains according to the different AST guidelines, namely CLSI M100, CLSI VET01S and EUCAST, is represented. The differences in AST evaluation results are highlighted.

		*E. coli* Total Strains (n = 74)			*E. coli* Strains from Pyometra-Presenting Dogs (n = 33)			*E. coli* Commensal Strains (n = 41)		
Drug/Interpretation (%)		M	V	EUC	M	V	EUC	M	V	EUC
	R	59.5	59.5	59.5	51.5	51.5	51.5	65.9	65.9	65.9
Ampicillin	I	18.9	18.9	0.0	27.3	27.3	0.0	12.2	12.2	0.0
	S	21.6	21.6	40.5	21.2	21.2	48.5	22.0	22.0	34.1
Amoxicillin	R	29.7	29.7	54.1	21.2	21.2	51.5	36.6	36.6	56.1
clavulanic acid	I	13.5	13.5	0.0	24.2	24.2	0.0	4.9	4.9	0.0
	S	56.8	56.8	45.9	54.5	54.5	48.5	58.5	58.5	43.9
	R	-	5.4	-	-	0.0	-	-	9.8	-
Enrofloxacin	I	-	6.8	-	-	3.0	-	-	9.8	-
	S	-	87.8	-	-	97.0	-	-	80.5	-
	R	16.2	16.2	10.8	18.2	18.2	15.2	14.6	14.6	7.3
Cephalexin	I	32.4	32.4	0.0	30.3	30.3	0.0	34.1	34.1	0.0
	S	51.4	51.4	89.2	51.5	51.5	84.8	51.2	51.2	92.7
	R	4.1	-	1.4	3.0	-	0.0	4.9	-	2.4
Cefotaxime	I	0.0	-	1.4	0.0	-	0.0	0.0	-	2.4
	S	95.9	-	97.3	97.0	-	100	95.1	-	95.1
Sulfamethoxazole	R	10.8	10.8	10.8	0.0	0.0	0.0	19.5	19.5	19.5
Trimethoprim	I	1.4	1.4	1.4	3.0	3.0	3.0	0.0	0.0	0.0
	S	87.8	87.8	87.8	97.0	97.0	97.0	80.5	80.5	80.5

Legend: M—CLSI M100; V—CLSI VET01S; EUC—EUCAST; R—resistant; I—intermediate; S—susceptible.

## Data Availability

The datasets used in the current study are available from the corresponding author on reasonable request.
